# *RYR1*-Related Myopathies Involve More than Calcium Dysregulation: Insights from Transcriptomic Profiling

**DOI:** 10.3390/biom15111599

**Published:** 2025-11-14

**Authors:** Daniele Sabbatini, Domenico Gorgoglione, Giovanni Minervini, Aurora Fusto, Matteo Suman, Chiara Romualdi, Sara Vianello, Giuliana Capece, Gianni Sorarù, Caterina Marchioretti, Maria Pennuto, Luca Vedovelli, Gyorgy Szabadkai, Luca Bello, Elena Pegoraro

**Affiliations:** 1 Neuromuscular Unit, Department of Neuroscience, University of Padova, 35128 Padova, Italy; daniele.sabbatini@unipd.it (D.S.); domenico.gorgoglione@studenti.unipd.it (D.G.); aurora.fusto@gmail.com (A.F.); sara.vianello@unipd.it (S.V.); giuliana.capece@studenti.unipd.it (G.C.); gianni.soraru@unipd.it (G.S.); luca.bello@unipd.it (L.B.); 2 Unit of Biostatistics, Epidemiology, and Public Health, Department of Cardiac, Thoracic, Vascular and Public Health Sciences, University of Padova, 35131 Padova, Italy; luca.vedovelli@unipd.it; 3 Department of Biomedical Sciences, University of Padova, 35131 Padova, Italy; giovanni.minervini@unipd.it (G.M.); matteo.suman@gmail.com (M.S.); caterina.marchioretti@unipd.it (C.M.); maria.pennuto@unipd.it (M.P.); gyorgy.szabadkai@unipd.it (G.S.); 4 Department of Biology, University of Padova, 35131 Padova, Italy; chiara.romualdi@unipd.it; 5 Venetian Institute of Molecular Medicine (VIMM), 35129 Padova, Italy; 6 Department of Cell and Developmental Biology, Consortium for Mitochondrial Research, University College London, London WC1E 6BT, UK

**Keywords:** skeletal muscle diseases, RYR1-RM, RYR1, CCD, MmD, RNASeq

## Abstract

Ryanodine receptor 1-related myopathies (RYR1-RM) are caused by *RYR1* gene variants and comprise a wide spectrum of histopathological manifestations. Here, we focus on patients carrying *RYR1* variants and muscle histopathology consistent with central core disease (CCD) or multi-minicore disease (MmD). RNA-sequencing analyses of skeletal muscle biopsies obtained from both CCD and MmD patients and from healthy controls were performed to better understand the molecular pathways activated by *RYR1* variants. Our analyses revealed that, beyond the well-established role of RYR1 in calcium homeostasis, broader cellular pathways are implicated. In CCD, differentially expressed genes were enriched for pathways related to oxidative stress response, SMAD signalling, and apoptosis, consistent with the role of intracellular calcium dysregulation in promoting mitochondrial dysfunction and cell death. In contrast, MmD patients exhibited enrichment of pathways related to immune activation. This was corroborated by the upregulation of GTPase-regulating genes and the down-regulation of transcriptional repressors such as *ZFP36* and *ATN1.* When considering all RYR1-RM patients collectively, Wnt signalling, immune-related pathways, and oxidative phosphorylation emerged as shared enriched pathways, indicating possible convergent mechanisms across histopathological phenotypes. Our study suggests that complex gene regulation driven by *RYR1* variants may be a unifying feature in CCD and MmD, offering new insight into potential therapeutic targets.

## 1. Introduction

Ryanodine receptor 1-related myopathies (RYR1-RM) are the most common non-dystrophic congenital myopathies with an estimated prevalence of 1 in 90,000 individuals [1]. They present with a wide range of histological and clinical manifestations [2,3]. In a subset of patients, skeletal muscle is characterised by the presence of “cores”—focal areas of reduced oxidative enzyme activity, absence of mitochondria, and variable degrees of myofibrillar disruption [4]. Based on muscle biopsy’s histological findings, RYR1-RM patients are most commonly classified as having either central core disease (CCD) or multi-minicore disease (MmD). CCD is typically associated with single, well-defined cores located in the centre of type 1 muscle fibers. In contrast, MmD is defined by multiple, less distinct cores scattered throughout the fibers, without fiber type specificity [5,6]. This binary classification is becoming increasingly inadequate. Both central cores and multi-minicores can coexist within the same biopsy, and features of other congenital myopathies often appear together with cores, and even a transition from the main histopathology features towards features of other congenital myopathies is common (i.e., rods) [7,8,9]. Moreover, new histopathological entities with modified core features, such as Dusty Core Disease (DuCD), have also been described [10], further expanding the histopathological spectrum of RYR1-RM. While CCD is primarily a monogenic disorder caused by variants in *RYR1*, MmD is genetically more heterogeneous and can be associated with variants in several other genes [3,11,12,13,14,15,16,17,18,19,20,21,22,23]. RyR1, the protein coded by *RYR1*, is the main calcium-release channel of the sarcoplasmic reticulum (SR) in skeletal muscle. Due to its pivotal role in excitation–contraction coupling, RyR1 activity is finely regulated by several post-translational modifications, as well as protein–protein and protein–ligand interactions [24]. This regulatory complexity, coupled with the diverse physiological roles of calcium signalling, gives rise to multiple potential pathomechanisms in RYR1-RM. These include: increased sensitivity of RyR1 channels to activators, enhanced RyR1-mediated calcium leak, and excitation–contraction uncoupling (i.e., impaired calcium release due to reduced RyR1 permeability and reduced RyR1 expression) [24,25].

As a result, genotype–phenotype correlations in RYR1-RM are complex. Dominant gain-of-function missense variants cluster within three hot spot regions: the N-terminal MHS/CCD region 1 (amino acids 35–614), the central MHS/CCD region 2 (amino acids 2163–2458), and the dominant RYR1 C-terminal region (amino acids 4550–4990, Figure S1) [6]. In contrast, recessively inherited variants associated with MmD, typically biallelic missense/truncating variants, are distributed outside these hot spots. They are primarily located within the SPRY, handle, and central solenoid regions, leading to reduced functional channel expression or ECC, and are often linked to earlier-onset congenital myopathies Figure S1 [6,26,27].

Clinical severity generally correlates with the mode of inheritance, consistent with findings that recessive cases tend to present with more severe phenotypes, particularly involving bulbar and respiratory muscles during the neonatal period. Hypomorphic variants have been associated with increased disease severity, and patients carrying at least one such allele typically exhibit more pronounced clinical manifestations [26].

To explore whether different molecular pathways are activated downstream of *RYR1* variants (potentially explaining the formation of central core versus minicores) and to better understand the molecular basis of the disease, we performed RNA-sequencing on skeletal muscle biopsies from CCD and MmD patients carrying *RYR1* variants. The goal was to characterise the transcriptomic profiles of these subgroups in comparison with healthy controls.

## 2. Materials and Methods

### 2.1. Sample and Ethics Statement

Skeletal muscle biopsies were obtained from fifteen patients with defined *RYR1* variants. All biopsies were obtained during routine clinical practice. Immediately after excision, each specimen was oriented and snap-frozen by immersion in the liquid phase of isopentane pre-cooled in liquid nitrogen, then stored at −80 °C until analysis. Based on histological evaluation (COX, SDH, and NADH stainings), the patients were categorised as CCD (*n* = 10) or MmD (*n* = 5). Seven histologically normal individuals served as controls (total *n* = 22). Among patients, 6 were male; in the control group, 4 were male. According to age at biopsy, 3 patients were paediatric (<18 years; ID_14, ID_15, ID_16) and 12 were adults. Mean age at biopsy was 30.9 ± 13.8 years (range 12–53) in CCD, 34.9 ± 13.9 years (range 6–55) in MmD, and 25.1 ± 20.1 years (range 3–57) in controls. Written informed consent was obtained from all individuals or their legal guardians, in accordance with the ethical standards of the 1975 Declaration of Helsinki (revised in 2013), prior to muscle biopsy collection.

### 2.2. RNA Extraction and Quantification

Fifty 30 µm thick slices were collected from each frozen skeletal muscle biopsy. Total RNA was extracted following the TRIzol™ (Invitrogen, Waltham, MA, USA; 15596026) protocol for RNA extraction, and RNA was reverse-transcribed using the SuperScript™ III Reverse Transcriptase (Thermo Fisher Scientific, Waltham, MA, USA; 18080085) following the manufacturer’s instructions. The extracted RNA was quantified using Qubit technology.

### 2.3. Sequencing

The QuantSeq 3′ mRNA-seq Library Prep kit (Lexogen; Vienna, Austria) was used for library construction. Reads preprocessing was performed using fastp v0.20.0 [28], applying specific parameters in order to remove residual adapter sequences and to keep only high-quality data (qualified_quality_phred = 20, unqualified_percent_limit = 30, average_qual = 25, low_complexity_filter = True, complexity_threshold = 30). Alignment was carried out using rsem v1.3.3 and STAR v2.7.0, against the Homo_sapiens.GRCh38.99.dna.primary_assembly.fa genome, downloaded from Ensembl. Each library was sequenced using single-end (SE) reads of 75 bp, producing an average of approximately 10.7 million reads per sample. About 86–88% of reads mapped uniquely to the human reference genome, yielding roughly 9 million informative reads per sample. The average per-base mismatch rate was below 0.6%, indicating high sequencing quality and sufficient depth for 3′ RNA-seq–based differential expression analyses. The annotation files (the transcript dataframe, the gene-level dataframe, and, subsequently, the tx2gene file) were obtained using the functions ensambldb [29] and AnnotationHub v3.14.0 (available as R/bioconductor packages). DESeq2 v1.46.0 [30] (available as an R/bioconductor package) was used for differential expression analysis by carrying out pairwise comparisons between CCD and controls and between MmD and controls (all details in Figure S2). To identify dysregulated genes in patients and explore the pathways underlying the pathology, RNA-seq was conducted on all 22 patients and controls. Sequencing was conducted for gene-level analysis. On average, 10,734,441 reads per sample were generated and processed using fastp v0.20.0 [28]. All samples passed quality control. The percentage of uniquely mapped reads was high, with a mean of 87%. After aligning to the reference genome, transcript abundance was calculated. Statistical testing was restricted to Ensembl protein-coding genes on standard chromosomes 1–22, X, Y, and MT; genes with zero counts across all samples were excluded, yielding 19,968 genes for DESeq2 analyses.

### 2.4. Differential Expression Analysis

DESeq2 fits a generalised linear model (GLM) for each gene by modelling the read count along a negative binomial distribution, considering the average and the dispersion.

The GLM fit returns coefficients indicating the overall expression level of the gene and the log_2_ fold change (log2FC) between the first and the second group considered [30]. This method considers ingroup variability, that is, variability among replicates, by modelling a dispersion parameter that describes the variance of counts (using maximum likelihood). This is pivotal in experiments in which dispersion estimates risk to be highly variable for each gene.

The statistical test implemented by DESeq2 is the Wald test, where the null hypothesis is set as log2FC = 0, representing no difference in expression. A Z-statistic is used for *p*-value calculation to determine whether to reject the null hypothesis. Values are then adjusted according to the Benjamini–Hochberg (HB) method into *p*-adjusted values (*p*-adj). As a filtering parameter, DESeq2 considers the average expression level of each gene across all samples and excludes all genes exhibiting mean normalised counts lower than a threshold after adjustment for multiple testing. We applied the experimental design, “design = ~ sex + age group + sample type”, with “sample type” labeling the comparison groups (e.g., CCD vs. Ctrl), to compare transcriptional profiles pairwise using sex and age group as covariates.

Successful normalisation (implemented by default by DESeq2) was verified by comparing the boxplots of the samples before and after such process using plotRLE in the package EDAseq v2.40.0 and counts function from BiocGenerics v0.52.0 (both available as R/bioconductor packages).

Differentially expressed genes (DEGs) were defined as those with a *p*-adj < 0.05 and log2FC greater than 0.58 or less than −0.58.

### 2.5. Principal Component Analysis

The sequencing results were used to compute principal components with the plotPCA function from BiocGenerics; data were first transformed with rlog, chosen for its ability to stabilise variance across a moderate number of samples, and then the top 10% most variable transcripts across the dataset were selected for the PCA. To quantify subgroup separation independently of covariates, we ran a group-only PERMANOVA (999 permutations) on Euclidean distances computed from rlog-transformed counts (same feature selection used for PCA). PERMANOVA is a non-parametric, distance-based ANOVA that tests whether between-group centroids differ more than expected by chance. We applied this procedure to four pre-specified contrasts (CCD vs. controls, MmD vs. controls, paediatric vs. adult, and male vs. female).

### 2.6. Functional Pathway Analysis

Two functional enrichment analyses were carried out. First, the over-representation analysis (ORA) considering the Gene Ontology (GO) terms established by the Gene Ontology project (http://geneontology.org/, accessed on 10 February 2025) through the function EnrichGO in clusterProfiler v4.14.6 [31]. This analysis is based on a hypergeometric test, which considers the probability of observing the proportion of differentially expressed genes in the functional category of choice over the total list of genes yielded by DE analysis, based on the proportion of genes involved in the category of choice over the whole human gene pool. The results in terms of enriched pathways were visualised via dotplot and their functional relationship via emapplot and cnetplot, the latter being functions in the package enrichplot v1.26.6 [32], available in Bioconductor.

Next, gene set enrichment analysis (GSEA) was carried out using clusterProfiler [31] and pathview v1.46.0 [33], considering the log_2_ fold-change values obtained through differential expression analysis relative to a whole gene set instead of those of the single genes. This analysis was carried out on KEGG gene sets (https://www.genome.jp/kegg/pathway.html, accessed on 10 February 2025). Data resulting from this analysis were integrated to visualise a schematic of the pathways using clusterProfiler. In particular, we used the “emaplot” function to display pathway relationships as a network, where nodes represent enriched terms and edges indicate functional similarity based on the overlap of associated genes. Distinct clusters in the emaplot reflect groups of pathways sharing similar gene sets, allowing for the identification of functionally related processes. Additionally, the “category netplot” to illustrate the connections between genes and pathways was used to provide a detailed view of which genes contribute to each enriched term.

### 2.7. Gene Validation

Gene expression was measured by qRT-PCR using the SsoAdvanced Universal Sybr Green Supermix (1725274; Bio-Rad; Hercules, CA, USA) and the C1000 Touch Thermal Cycler-CFX96 Real-Time SYSTEM (Bio-Rad). The list of specific primers (Eurofins Scientific; Luxembourg City, Luxembourg) is provided in Table S1. Gene transcript levels were normalised to actin. The results were then statistically analysed with the Wilcoxon Mann–Whitney test.

## 3. Results

### 3.1. Patient Cohort

In this study, 15 participants were evaluated, as detailed in Table 1. The cohort consisted of 10 patients diagnosed with CCD and 5 with MmD. Seven muscle biopsies from healthy subjects were used as controls. Within the CCD subgroup, there were eight female patients, two of whom were 12 years old and classified as paediatric patients. The MmD group included four adults (one female) and one 6-year-old male, who was categorised as a paediatric patient. All *RYR1* variants have been previously described [3]. Patient ID_01 and patient ID_05 were father and son, respectively. Detailed clinical information has been previously reported [3]. Among the 15 affected individuals analysed, clinical manifestations were heterogeneous but predominantly mild. A positive family history consistent with autosomal dominant inheritance was identified in 8/15 of cases, whereas the remainder were apparently sporadic. Foetal hypokinesia and arthrogryposis were absent, indicating normal early motor development in the majority of subjects. Two individuals had a floppy infant presentation, but only one progressed to a severe myopathy. Independent ambulation was typically achieved within the expected age range. Muscle weakness was present in six patients and was mild in three (Table S2). Respiratory insufficiency and dysphagia were absent, reflecting limited involvement of the respiratory and bulbar musculature. Ocular manifestations were uncommon, with only one isolated case of ophthalmoparesis. Contractures and foot deformities represented the most frequent musculoskeletal abnormalities, while scoliosis was observed in approximately half of the cohort. No evidence of respiratory or cardiac involvement was identified, and cognitive function was preserved in all patients. Serum creatine kinase (CK) values were mostly within normal limits, though occasional mild to moderate elevations were detected (Table S2).

In our cohort, four patients carried compound heterozygous *RYR1* variants: only in patient ID14 was segregation analysis consistent with a recessive inheritance. In the other three patients, relatives were not available. In patient ID_06, the p.Pro1787Leu variant was previously reported as benign [39].

### 3.2. Differentially Expressed Genes (DEGs)

**Figure 1:**
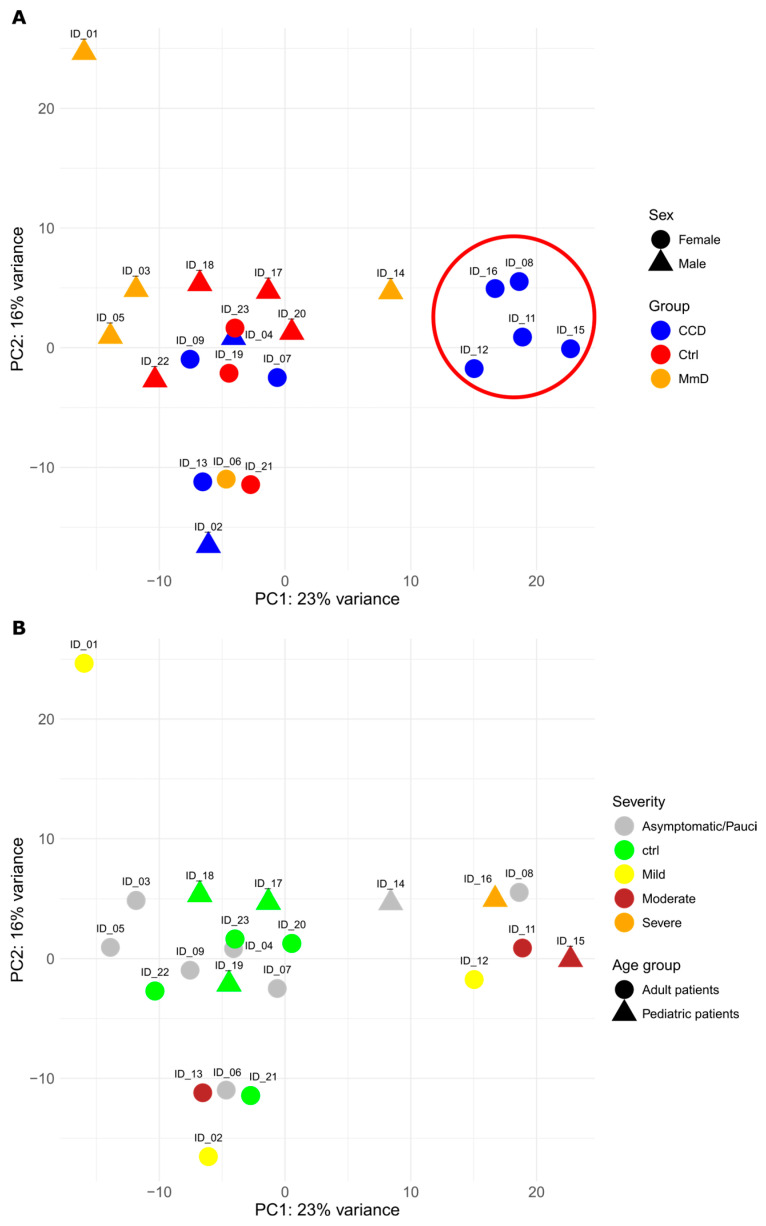
(**A**) The graph displays patients described by the first two principal components, which account for 37% of the variability. CCD patients are represented in blue, MmD patients in orange, and healthy controls in red. Sex groups are differentiated by geometric shapes. A subgroup of female CCD patients appears to cluster distinctly within the PCA plot, as highlighted by the encircled region. (**B**) PCA with alternative colour coding (severity and age group). The graph displays patients described by the first two principal components, which account for 37% of the variability. Age groups are differentiated by geometric shapes, with paediatric patients (<12 years) and adult patients (≥12 years) represented by distinct markers. The colours correspond to the severity of the disease: grey for asymptomatic individuals, green for healthy controls, yellow for mild myopathy, brown for moderate myopathy, and orange for severe myopathy.

From the initial pool of transcripts, the top 10% (1997) were subjected to rlog transformation for principal component analysis (PCA). PCA was used as a descriptive QC visualisation (Figure 1A and Figure S3) and did not show clearly separated clusters of RYR1-RM patients (both CCD and MmD) from the control group. However, a subgroup of five female CCD patients (referred to as CL5 CCD) displays a distinct transcriptional signature, highlighted by the encircled region. This pattern appears independent of age or disease severity, as further illustrated in Figure 1B. Moreover, a subset of MmD patients (specifically IDs 03, 05, and 01) is predominantly located on the left side of the PCA plot, suggesting potential divergence within the MmD group along the first principal component. As a result, the analysis was extended to assess these sub-phenotypic groups (CCD and MmD), with a particular focus on the CL5 CCD subgroup, to further elucidate the underlying transcriptional differences. To quantify subgroup separation independently of covariates, we performed a PERMANOVA on Euclidean distances computed from rlog-transformed counts. The analysis indicated a modest but significant separation for CCD vs. controls (R^2^ = 0.145, *p* = 0.010), no robust evidence of separation for MmD vs. controls (R^2^ = 0.106, *p* = 0.230), a small but detectable effect for paediatric vs. adult (R^2^ = 0.095, *p* = 0.010), and a modest, significant effect for male vs. female (R^2^ = 0.110, *p* = 0.006). These results motivated the inclusion of sex and age as covariates in subsequent analyses to account for their contribution to between-sample dispersion.

#### 3.2.1. CCD Versus Controls

**Figure 2:**
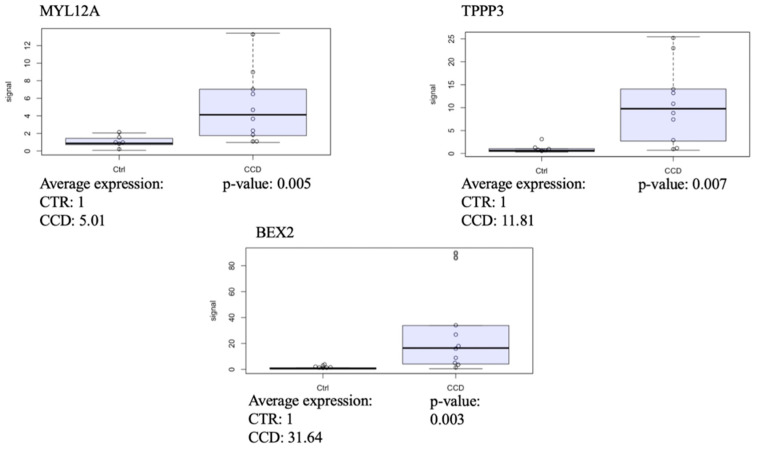
RT–PCR analysis of the transcript levels of the indicated genes normalised to beta-actin transcript levels in the CCD muscle (*n* = 10) compared with controls (*n* = 6).

**Figure 3:**
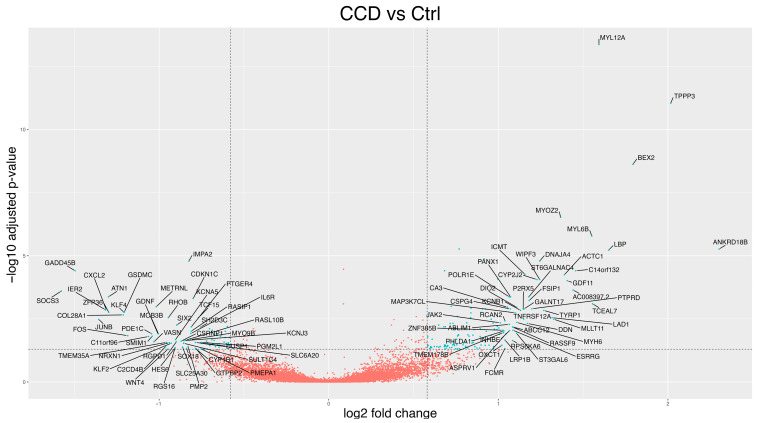
Differential expression analysis of CCD vs. controls using DESeq2. Each dot represents a gene, plotted according to its log2FC on the X-axis and the −log10 adjusted *p*-value on the Y-axis. Blue dots indicate DEGs (*p*-adj < 0.05 with a log2FC ≥ 0.58 or ≤−0.58). DEGs were labeled (only a subset of the most dysregulated genes was shown for readability).

In this section, we first performed a transcriptional profile analysis comparing all CCD patients to controls, adjusted for sex and age, using DESeq2. This analysis identified 221 differentially expressed genes (DEGs, *p*-adj < 0.05 and log2FC greater than 0.58 or less than −0.58), of which 143 were up-regulated (Figure 2, Table S3). We then conducted an in silico validation analysis focusing on the subgroup of patients, referred to as CL5 CCD, who exhibited a distinct gene expression pattern based on principal component analysis. Comparing the transcriptional profile of CL5 CCD patients to controls, we identified 735 DEGs. Of these, 367 were up-regulated and 368 down-regulated (Table S4). This step aimed to assess the consistency of DEGs between the full CCD cohort and the CL5 CCD subgroup, in order to determine whether the more pronounced transcriptional changes observed in the CL5 patients were representative of the broader CCD group. Among the 160 DEGs shared between the two analyses (all CCD vs. controls and CL5 CCD vs. controls), the top three in terms of statistical significance were as follows: myosin light chain 12A (*MYL12A*, *p*-adj = 4.32 × 10^−14^, log2FC = 1.591 and *p*-adj = 1.06 × 10^−26^, log2FC = 2.05, respectively), tubulin polymerisation promoting protein family member 3 (*TPPP3*, *p*-adj = 9.07 × 10^−12^, log2FC = 2.01 and *p*-adj = 6.00 × 10^−28^, log2FC = 2.93), and brain expressed X-linked 2 (*BEX2*, *p*-adj = 2.41 × 10^−09^, log2FC = 1.79 and *p*-adj = 5.00 × 10^−12^, log2FC = 3.25). These genes are potentially involved in mitochondrial dynamics, promoting mitochondrial fission [48] and mitophagy [49], although their precise roles remain to be fully elucidated. Validation via RT-qPCR confirmed their differential expression (*p* = 0.005, 0.007, and 0.003, respectively, Figure 3). The findings reinforced the consistency between the two analyses and support the hypothesis that the CL5 CCD subgroup is representative of the broader CCD population.

Among the genes with statistically significant changes in expression, suppressor of cytokine signalling 3 (*SOCS3*, *p*-adj = 0.0002, log2FC = −1.58), growth arrest and DNA damage inducible beta (*GADD45B*, *p*-adj = 3.92 × 10^−5^, log2FC = −1.50), and JunB proto-oncogene, AP-1 transcription factor subunit (*JUNB*, *p*-adj = 0.003, log2FC = −1.36) exhibited the strongest down-regulation. These genes were also significantly down-regulated in the analysis restricted to the CL5 CCD subgroup (Table S4), further supporting their potential involvement in the disease-specific transcriptional signature. The most down-regulated genes are possibly involved in mitochondrial stress, reducing mitochondrial membrane potential [50] or inhibiting mitochondrial biogenesis by reduced activation of the p38 MAPK pathway [51].

#### 3.2.2. MmD Versus Controls

**Figure 4:**
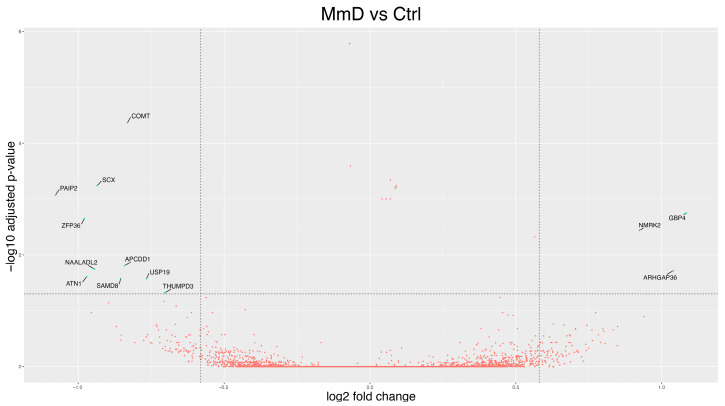
Volcano plot showing the results of differential expression analysis of MmD vs. controls using DESeq2. Each dot represents a gene, plotted according to its log2FC on the *X*-axis and the −log10 adjusted *p*-value on the *Y*-axis. Blue dots indicate DEGs (*p*-adj < 0.05 with a log2FC ≥ 0.58 or ≤−0.58). DEGs were labeled.

Differential expression analysis comparing the 5 MmD patients to controls identified 13 DEGs (*p*-adj < 0.05 and log2FC change ≥ 0.58 or ≤−0.58), with 3 upregulated and 10 down-regulated (Figure 4, Table S5). Among the DEGs, the most strongly down-regulated were the transcription repressor poly(A) binding protein interacting protein 2 *(PAIP2*; *p*-adj = 0.00085, log2FC = −1.08), zinc finger protein 36 (*ZFP36*; *p*-adj = 0.0022, log2FC = −0.98), and atrophin 1 (*ATN1*; *p*-adj = 0.0247, log2FC = −0.97). Down-regulation of *ZFP36* may result in a more stable PGC-1α mRNA, leading to increased PGC-1α protein levels and improved mitochondrial function [52]. Among the most up-regulated DEGs, we identified different GTPase-related genes, including guanylate-binding protein 4 (*GBP4*, *p*-adj = 0.002, log2FC = 1.08) and Rho GTPase activating protein 36 (*ARHGAP36*, *p*-adj = 0.02, log2FC = 1.04). The upregulation of *ARHGAP36* was confirmed by qRT-PCR (Figure S4).

#### 3.2.3. All RYR1-RM Patients vs. Controls

Finally, we conducted a DEG analysis considering all RYR1-RM patients compared to controls (Figure S5). In total, 47 DEGs (*p*-adj < 0.05 with a log2FC ≥ 0.58 or ≤−0.58) were identified, consisting of 27 upregulated and 20 down-regulated genes (Table S6). Among the most differentially expressed genes, several had already been identified in the subgroup analyses. Notably, *ZFP36* (*p*-adj = 3.26 × 10^−7^, log2FC = −1.57) and *ATN1* (*p*-adj = 7.25 × 10^−4^, log2FC = −1.18) emerged as the most down-regulated genes, and were the only ones consistently identified in both the CCD vs. controls and MmD vs. controls comparison. Among the DEGs, the most strongly upregulated were A*NKRD18B* (*p*-adj = 2.56 × 10^−4^, log2FC = 1.08), *TPPP3* (*p*-adj = 9.13 × 10-03, log2FC = 0.97), followed by *MYL12A* (*p*-adj = 0.011, log2FC = 0.94). In addition, we detected *ARHGAP36* (*p*-adj = 0.039, log2FC = 0.77), consistent with the MmD vs. controls comparison.

#### 3.2.4. Symptomatic-Only Analyses

To enrich the analysis of DEGs between patients and controls, we performed a transcriptional profiling analysis, including only symptomatic patients defined as those presenting with muscle weakness. Pooled symptomatic RYR1-RM (*n* = 7) vs. controls (*n* = 7) identified 181 DEGs (*p*-adj  <  0.05 and log2FC ≥ 0.58 or ≤−0.58), 93 up and 88 down (Figure S6, Table S7). Some genes identified in the original analyses remained robust: *TPPP3* (*p*-adj = 1.16 × 10^−15^, log2FC  =  2.08), *BEX2* (*p*-adj = 8.48 × 10^−7^, 1.61), and *MYL12A* (*p*-adj = 7.59 × 10^−3^, 1.11) were up-regulated. Notably, *SOCS3* (2.12 × 10^−5^, −1.69), *GADD45B* (7.59 × 10^−3^, −1.22), and *JUNB* (8.81 × 10^−3^, −1.37) now emerged as down-regulated, as also observed in the CCD-versus-control comparison. Consistent with subgroup findings, *ZFP36* (*p*-adj = 4.35 × 10^−8^, −2.06) and *ATN1* (*p*-adj = 1.36 × 10^−5^, −1.54) ranked among the most significant DEGs. Two additional pooled signals also reached significance: *ARHGAP36* (*p*-adj = 0.0226, 1.19) and *PAIP2* (*p*-adj = 0.0378, −0.77). For the symptomatic CCD (*n* = 6) vs. controls (*n* = 7), there were 380 DEGs (*p*-adj  <  0.05 and log2FC ≥ 0.58 or ≤−0.58; 182 up, 198 down; Figure S7, Table S8). *TPPP3* (*p*-adj = 6.00 × 10^−19^, 2.39), *BEX2* (*p*-adj = 5.79 × 10^−11^, 2.42), and *MYL12A* (*p*-adj  =  1.47 × 10^−11^, 1.64) were up-regulated, and *SOCS3* (*p*-adj =  3.31 × 10^−6^, −2.06), *GADD45B* (*p*-adj  =  6.89 × 10^−4^, −1.51), and *JUNB* (*p*-adj =  9.97 × 10^−4^, −1.68) down-regulated. *ZFP36* (*p*-adj  =  3.57 × 10^−10^, −2.57) and *ATN1* (*p*-adj = 1.46 × 10^−4^, −1.54) showed patterns consistent with previous analyses. Only one symptomatic MmD patient was available and was compared to controls (*n* = 7). In this case, 221 (115 up, 106 down) DEGs were identified (*p*-adj  <  0.05 and log2FC ≥ 0.58 or ≤−0.58, Figure S8, Table S9). The MmD transcriptional signature was confirmed: *ARHGAP36* (*p*-adj = 1.65 × 10^−3^, log2FC = 3.47) was up-regulated, while *ATN1* (*p*-adj = 1.51 × 10^−2^, −1.82) and *PAIP2* (*p*-adj = 5.31 × 10^−4^, −1.66) were down-regulated. *MYL12A* showed significant down-regulation (*p*-adj = 7.50 × 10^−5^, −1.21). Stress-response genes (*SOCS3*, *GADD45B*, *JUNB*) and *ZFP36* did not reach significance in this analysis. Among the most extreme changes, the top up-regulated DEGs were *JCHAIN* (*p*-adj = 4.38 × 10^−38^, log2FC = 6.38) and *CCL13* (*p*-adj = 6.44 × 10^−13^, 4.09), while the top down-regulated were *TECRL* (*p*-adj  =  1.75 × 10^−9^, −4.17) and *IDI2* (*p*-adj =  2.92 × 10^−19^, −3.50).

### 3.3. Analyses of Gene Function

Analyses of gene function were conducted by both over-representation analysis (ORA) using Gene Ontology (GO) terms and by Gene Set Enrichment Analysis (GSEA) using KEGG ontology gene sets.

#### 3.3.1. Gene-Function Analyses: CCD Versus Controls

**Figure 5:**
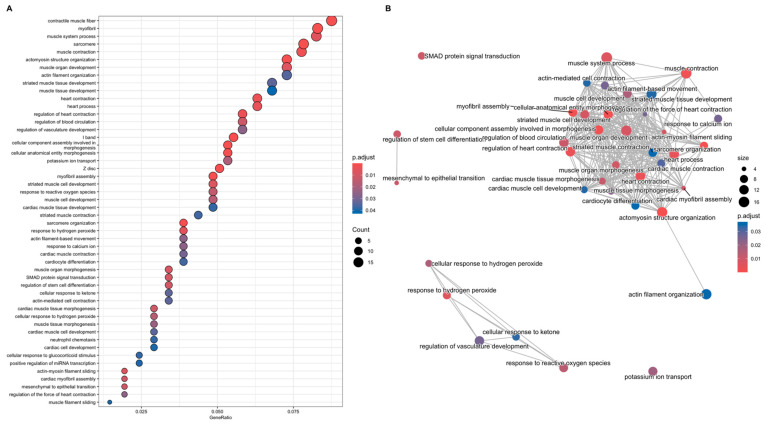
(**A**) Dotplot showing the top 50 Gene Ontology (GO) terms according to the gene ratio (DEGs related to a GO term/total number of genes of the GO term). The colours represent the *p*-value adjusted according to the HB method (*p*-adj, as in the legend), and the dots’ size indicates the number of genes involved in each pathway. (**B**) Dotplot showing existing relationships among the 50 most significant enriched GO terms (*p*-adj) resulting from over-representation analysis. For graphical reasons, not all labels corresponding to the muscle function and muscle physiology macrocategory are displayed.

**Figure 6:**
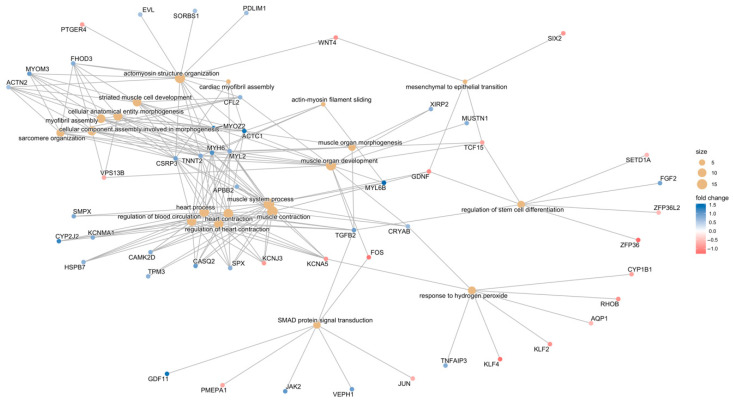
Relationships occurring between genes associated with the 20 most significant GO terms and relative fold change (colour). The size of the dots indicates the number of genes involved in each pathway.

DEGs identified in the CCD vs. control comparison were analysed using GO-ORA, identifying 62 significantly enriched GO terms (Figure 5A, Table S10). The most significantly enriched pathways were primarily related to muscle function and physiology. Interestingly, several unexpected pathways also emerged, including response to reactive oxygen species, SMAD protein signal transduction, response to hydrogen peroxide, and positive regulation of miRNA transcription. Notably, these pathways form a distinct cluster, as visible in the emaplot generated for the 50 most significant GO terms (Figure 5B). The relationships among genes within each pathway are illustrated in the category netplot (Figure 6).

GSEA using KEGG gene sets identified 33 significantly enriched pathways (*p*-adj < 0.05), among which the apoptosis pathway was particularly notable (*p*-adj = 0.018, and contributing 31% of the enrichment signal, as shown in Table S11).

#### 3.3.2. Gene-Function Analyses: MmD Versus Controls

In the MmD vs. control comparison, no GO terms were significantly enriched in the ORA. Conversely, GSEA-KEGG identified 55 significantly enriched pathways (*p*-adj < 0.05), outlining a coherent immune–metabolic signature (Table S12). Among the most significant, we found antigen processing and presentation, *Staphylococcus aureus* infection, autoimmune thyroid disease, graft-versus-host disease, oxidative phosphorylation, natural killer cell-mediated cytotoxicity, and intestinal immune network for IgA production, with enrichment signals of approximately 37%, 35%, 43%, 44%, 40%, 34%, and 37%, respectively.

These pathways highlight a prominent immune and inflammatory component, involving antigen presentation, immunoglobulin A (IgA) production, NK-cell cytotoxicity, and allograft/autoimmune responses, together with a mitochondrial and metabolic axis represented by oxidative phosphorylation. This integrated profile suggests that immune activation and mitochondrial adaptation are central features of the MmD transcriptional landscape.

#### 3.3.3. Gene-Function Analyses: All RYR1-RM Patients Versus Controls

In the pooled RYR1-RM vs. controls analysis, GO-ORA identified five significantly enriched GO terms, forming a compact developmental cluster comprising renal vesicle morphogenesis, renal vesicle development, mesenchymal-to-epithelial transition, cell differentiation involved in metanephros development, and metanephric nephron morphogenesis (*p*-adj ≈ 0.03–0.04; fold-enrichment between 30× and 43×, Figures S9 and S10, Table S13). GSEA-KEGG identified 29 significantly enriched pathways (*p*-adj < 0.05), including oxidative phosphorylation, Wnt signalling, pathways in cancer, IL-17 signalling, breast cancer, diabetic cardiomyopathy, Huntington disease, allograft rejection, cardiac muscle contraction, and 2-oxocarboxylic acid metabolism, with enrichment signals of approximately 47%, 35%, 24%, 28%, 26%, 37%, 34%, 31%, 34%, and 46%, respectively.

The overall pattern highlights both metabolic and developmental components, with oxidative phosphorylation and cardiac muscle contraction pointing to mitochondrial activity and energy regulation, while Wnt and IL-17 signalling reflect broader regulatory and inflammatory processes. Notably, there is a substantial overlap with the MmD results, with 20 KEGG pathways shared between the two analyses (including antigen processing and presentation, allograft rejection, IL-17 signalling, insulin signalling, oxidative phosphorylation, and Wnt signalling), 12 were shared between the all-RYR1-RM and CCD analyses, and 6 were common to all 3 (oxidative phosphorylation, cardiac muscle contraction, wnt signalling pathway, IL-17 signalling pathway, pathways in cancer, breast cancer), underscoring common molecular mechanisms underlying the RYR1-RM disease spectrum (Table S14).

#### 3.3.4. Symptomatic-Only Gene-Function Analyses

The exclusion of asymptomatic/paucisymptomatic individuals substantially amplified pathway-level signals across controls.

In the first comparison, CCD vs. controls, GO-ORA now yields 182 significant GO terms at *p*-adj < 0.05 (Table S15), still dominated by muscle-structure/contractile programs (e.g., contractile muscle fiber, *p*-adj = 6.9 × 10^−10^; myofibril, 5.3 × 10**^−^**^9^; sarcomere, 1.9 × 10**^−^**^8^, Figures S11 and S12, Table S15). The oxidative-stress branch remains present (response to reactive oxygen species, 1.93 × 10^−3^; response to hydrogen peroxide, 3.61 × 10^−2^), whereas SMAD signal transduction does not reach *p*-adj < 0.05. KEGG GSEA identifies 116 significant pathways (Table S16), including oxidative phosphorylation (*p*-adj = 5.07 × 10^−6^) and apoptosis (2.05 × 10^−3^). Wnt signalling remains significant (*p*-adj = 1.19 × 10^−2^, Table S16). In the MmD vs. controls comparison, GO-ORA reports 431 significant GO terms (Figures S13 and S14, Table S17). KEGG GSEA increased from 54 to 97 significant pathways (Table S18), reinforcing the immune/infectious profile: *Staphylococcus aureus* infection (*p*-adj = 1.62 × 10^−9^), antigen processing and presentation, chemokine/cytokine–cytokine receptor signalling (both *p*-adj = 1.62 × 10^−9^), intestinal immune network for IgA production (*p*-adj = 1.62 × 10^−9^), phagosome (*p*-adj = 1.62 × 10^−9^), and cell-adhesion molecules (*p*-adj = 1.62 × 10^−9^). Finally, when considering all symptomatic RYR1-RM patients versus control returns, 11 significant GO terms were found (Figures S15 and S16, Table S19), mostly muscle development/contractile categories (muscle organ development, *p*-adj = 2.76 × 10^−4^; contractile muscle fiber, *p*-adj = 1.69 × 10^−3^; myofibril, *p*-adj = 3.20 × 10^−3^). KEGG GSEA yields 24 significant pathways (Table S20), with oxidative phosphorylation (*p*-adj = 1.89 × 10^−3^), Wnt signalling (*p*-adj = 9.90 × 10^−3^), and allograft rejection (*p*-adj = 9.90 × 10^−3^) remaining significant; *Staphylococcus aureus* infection also reaches significance here (*p*-adj = 2.68 × 10^−2^). Across contrasts, symptomatic-only analyses increase the number and strength of enriched pathways while preserving the directionality seen previously—i.e., muscle/contractile and oxidative-stress signatures in CCD, and immune/infectious/antigen-presentation programs in MmD—while the pooled symptomatic set retains oxidative phosphorylation, Wnt, allograft rejection, and gains *Staphylococcus aureus* infection.

### 3.4. Analysis of RYR1 Expression

**Figure 7:**
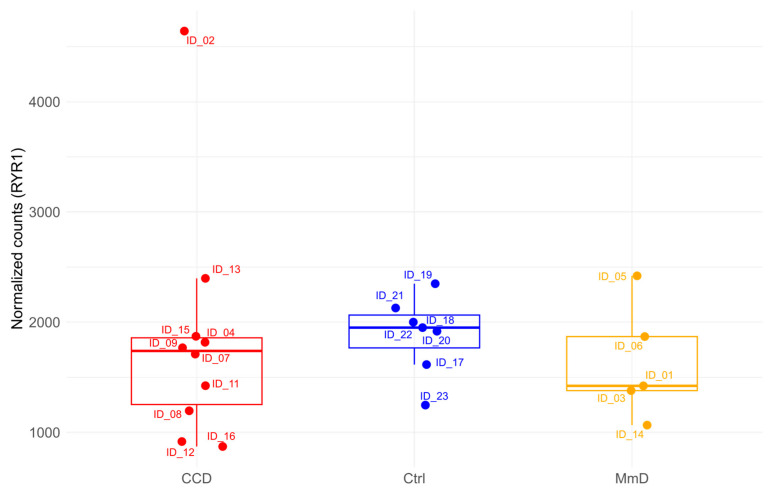
Normalised *RYR1* gene count distribution across phenotypic subgroups. The data are presented as boxplots indicating the distribution of normalised *RYR1* counts. Each observation is plotted as a point within its respective group: patients with CCD in red, Ctrl in blue, and MmD in yellow. The *y*-axis denotes the normalised counts for *RYR1*, facilitating a visual comparison of expression differences among the groups.

To quantify *RYR1* gene expression from our RNA-Seq dataset, we used the plotCounts function from the DESeq2 package to visualise normalised count data. This method applies the median-of-ratios approach to normalise read counts, adjusting for sample-specific size factors. Patient samples were stratified by histopathological phenotypic subgroups (CCD and MmD) to allow for subgroup-specific analysis of *RYR1* expression patterns (Figure 7). No statistically significant difference in RYR1 transcript levels was observed between groups (Wilcoxon test, *p* > 0.05).

Notably, within the CCD subgroup, patient ID_12, who carries a frameshift variant (p.Gln4837ArgfsX3), exhibited the lowest normalised *RYR1* expression. Similarly, in the MmD subgroup, patient ID_14, harbouring two variants (one predicted null, p.Ile1571Val and p.Leu3136ArgfsX3), also showed markedly reduced expression levels. These observations are consistent with the known biological effects of frameshift mutations, which often lead to truncated, non-functional proteins and can trigger mRNA degradation mechanisms such as nonsense-mediated decay.

## 4. Discussion

In this study, we conducted transcriptome-wide profiling of skeletal muscle biopsies from patients with RYR1-RM, including CCD and MmD, characterised at the histopathological level by a virtual absence of mitochondria and sarcoplasmic reticulum in the core regions. Our analysis revealed that, beyond the well-established role of RYR1 in calcium homeostasis, broader cellular pathways are likely implicated in disease pathogenesis.

### 4.1. Transcriptomic Distinction Between CCD and MmD

Principal component analysis revealed partial overlap of patient samples with controls but also showed a distinct separation between CCD and MmD along principal component 1 (Figure 1). This separation was more evident in the CCD subgroup, particularly among five females (CL5 CCD), than in the MmD group. We attempted to dissect the reasons underlying this clustering. Disease burden may be a contributing factor since all patients but one in the CL5 CCD cluster presented with a myopathy ranging in severity from mild to severe, likely enriching the gene dysregulation. Another tempting hypothesis is the possible sex-related effect. Even if both CCD and MmD are autosomal dominant or recessive diseases, estrogen and androgen receptors are expressed in skeletal muscle, and they are known to be able to modulate calcium signalling, oxidative phosphorylation, and muscle fiber type composition [53,54], thus possibly affecting the downstream consequences of RYR1 variants. Further studies are needed to underpin the relevance of these contributing factors.

### 4.2. Pathway Dysregulation in CCD: Muscle Function and Beyond

The over-representation analysis using Gene Ontology terms (ORA) for CCD revealed 62 dysregulated pathways, most of which were related to muscle structure and function (i.e., muscle contraction, muscle system process, striated muscle contraction, actin–myosin filament sliding, regulation of heart contraction, sarcomeric proteins, as shown in Table S10). These results are not surprising considering that in CCD, the core area and the area surrounding the cores are characterised by a focal myofibrillar degeneration and streaming or disintegration of the Z disks [55,56]. These findings also align with known consequences of *RYR1* variants, as previously observed in Ryr1 knock-out mouse models [57]. Besides the known deranged myofibrils, degenerating mitochondria or swollen mitochondria are often detected in the area surrounding the cores [56], and an increase in intracytoplasmic calcium can well explain the dysregulation of the oxidative stress response, response to reactive oxygen species (ROS), and response to hydrogen peroxide observed. The effects of specific *RYR1* variants have also been explored in several knock-in mouse models that reproduce malignant hyperthermia (MH) [41,58,59]. Across these models, mitochondrial dysfunction, most notably a reduction in oxidative-phosphorylation capacity, emerges as a recurrent hallmark and is thought to influence the MH contracture phenotype [60,61,62,63,64]. Complementing these pre-clinical data, a recent transcriptomic analysis of skeletal-muscle biopsies from malignant hyperthermia-susceptible (MHS) individuals showed that approximately 75% of differentially expressed genes were down-regulated and significantly enriched for oxidative-phosphorylation and fatty-acid-metabolism pathways [65]. Although follow-up protein studies will be required to confirm the functional impact of these transcriptional changes, the findings emphasise the importance of mitochondrial metabolism in human MH. Taken together with our results in CCD, they suggest that impaired electron-transport-chain activity is a common downstream consequence of RYR1 dysfunction.

This body of evidence aligns with electron microscopy observations in human samples but does not account for the absence of mitochondria in the core regions. A preliminary but interesting observation was the detection of dysregulated SMAD protein signalling [66,67].

SMAD signalling represents an emerging area of research in mitochondrial fusion and fission—fundamental processes governing mitochondrial network dynamics. These mechanisms determine the spatial distribution of mitochondria within cells and critically influence bioenergetics, metabolism, and functional efficiency. Smad2 facilitates mitochondrial fusion by scaffolding mitofusin 2 and enhancing its cytoplasmic fusion activity [68]. In contrast, Smad3 activation and phosphorylation, downstream of TGF-β signalling, promote mitochondrial fission through the AMPK pathway and other mitochondrial regulatory proteins [68]. Collectively, Smad proteins constitute integral modulators of mitochondrial dynamics, balancing fusion and fission events to maintain metabolic homeostasis and ensure appropriate cellular adaptation to stress or disease conditions.

Notably, although the canonical MAPK signalling pathway itself did not emerge as an enriched GO term, several key MAPK-related genes were differentially expressed [69,70,71], which are Ca^2+^-sensitive and implicated in mechanosensitive signalling [72]. These results suggest that *RYR1* variants may influence a broader range of cellular processes, including mitochondrial dynamics. Given the TGF-β/SMAD/MAPK pathways’ involvement in mitochondrial fission [73], it is plausible that altered mitochondrial network remodelling contributes to core formation.

### 4.3. Evidence for Apoptosis in CCD

GSEA using KEGG pathways identified apoptosis among the dysregulated pathways in CCD. Although apoptosis has not been widely reported in RYR1-RM, it is a common process in muscle pathology, including dystrophies, metabolic and mitochondrial myopathies, and inflammatory conditions [74].

Recent studies demonstrated increased cell death in RyR1^−/−^ and RyR1^+/−^ myotubes [75], attributed to ER stress activation [76,77] of the unfolded protein response (UPR) due to the elevated cytosolic Ca^2+^ levels. In our cohort, 24 DEGs overlapped with those reported in UPR-related gene expression following tunicamycin or thapsigargin treatment [78]. However, classical UPR markers were absent [79], preventing definitive conclusions about UPR involvement in CCD. Still, the role of apoptosis deserves further investigation. Several of the most upregulated genes in CCD suggest a pro-apoptotic signature: *TPPP3*, involved in microtubule dynamics, also regulates β-catenin/NF-κB/COX2 signalling and mitochondria-dependent apoptosis [80,81]; *BEX2*, which modulates NF-κB and c-Jun (*JNK* and *ErbB2* pathways, which are known to regulate mitochondrial apoptosis and G1-phase cell-cycle progression) [82,83]; and *MYL12A*, a regulatory myosin light chain, may suppress anti-apoptotic AATF/Che-1 and promote p53-mediated apoptosis (it also plays a role in actomyosin contractility and DNA damage response).

The most down-regulated DEGs -*SOCS3*, *GADD45B*, and *JUNB*- are also linked to apoptosis [84,85,86] and ER stress. For example, SOCS3 regulates GRP78 degradation and ER stress-induced apoptosis in cardiomyocytes [87,88], while JUNB protects cells from cytokine-induced cell death and modulates ER stress responses. It was unexpected that *MYL12A* emerged as the most overexpressed gene in our analysis, as it encodes a myosin regulatory light chain typically involved in smooth muscle and non-muscle cell contraction. Interrogation of the GTEx database confirmed that skeletal muscle exhibits the highest *MYL12A* expression level (dbGaP Accession phs000424.v10.p2), consistent with our RT-PCR results. A potential caveat is the heterogeneous composition of muscle biopsies, which include not only muscle fibers but also connective tissue, endothelial cells, small nerve bundles, and, occasionally, disease-associated infiltrating cells. However, CCD biopsies rarely display fibro-fatty replacement, and in our cohort, only 5 of 15 patients showed a mild increase in perimysial connective tissue, with no inflammatory infiltrates. Importantly, the strong *MYL12A* expression observed in GTEx was also evident at the single-cell level, supporting a relevant, although not yet known, role for *MYL12A* in skeletal muscle.

### 4.4. Transcriptomic Profile of MmD and Shared Mechanisms

In MmD patients, the small DEG set limited GO-ORA sensitivity (no significant GO terms), whereas KEGG GSEA revealed 55 dysregulated pathways (*p*-adj < 0.05). Several pathways overlapped with CCD, notably, Wnt signalling, IL-17 signalling, *Staphylococcus aureus* infection, and cell-adhesion molecules (Supplementary Tables S21 and S22 list the pathways discussed above together with side-by-side comparisons across analyses). Others were more MmD-specific, including antigen processing and presentation, intestinal immune network for IgA production, NK-cell–mediated cytotoxicity, and phagosome, together outlining an immune/infectious signature alongside a mitochondrial/metabolic axis (oxidative phosphorylation). At the gene level, key down-regulated DEGs included the transcriptional repressors *PAIP2* and *ATN1*, and *ZFP36*, an AU-rich-element RNA-binding protein involved in mRNA turnover, inflammation, and NF-κB regulation [89,90,91]. Among the upregulated genes, *ARHGAP36* and *GBP4* emerged as the most strongly upregulated transcripts. At this stage, the inflammatory signature identified in MmD cannot be fully interpreted. A re-examination of the MmD muscle biopsies, prompted by this transcriptomic finding, did not reveal histological evidence of inflammation. Specifically, no inflammatory infiltrates or even sparse lymphomonocytic cells were detected in the endomysial or perimysial connective tissue. These results suggest that the degree of immune activation in MmD is minimal and that the transcriptomic profile may reflect subtle or secondary immune-related processes rather than overt inflammation. It should be emphasised that RNA-seq is a discovery-driven rather than hypothesis-driven approach. By providing an unbiased and comprehensive overview of gene expression, RNA-seq allows the identification of unanticipated molecular signatures, including those related to immune regulation, stress responses, or metabolic adaptation. Consequently, the detection of an inflammatory signature in MmD may represent a downstream response to altered cellular homeostasis or mitochondrial dysfunction, rather than a primary immunopathological event. In contrast, and more consistent with observation in CCD, the cluster of dysregulated genes involved in translational control and mRNA regulation points to profound reorganisation of protein synthesis machinery and post-translational regulation. Such alterations are indicative of deep molecular remodelling in core myopathies, potentially reflecting adaptive mechanisms to disrupted energy metabolism, impaired calcium handling, or structural instability within the sarcomere. Together, these findings highlight the power of transcriptomic profiling to reveal unexpected aspects of disease biology and provide a foundation for future hypothesis-driven functional studies.

### 4.5. Symptomatic-Only Analysis—Rationale and Interpretation

In the attempt to enrich the sensitivity of our analyses, in line with the PCA, where many asymptomatic/paucisymptomatic cases clustered close to healthy controls and symptomatic cases tend to cluster together (CL5 CCD), we ran a complementary sensitivity analysis restricted to symptomatic patients (mild–severe), retaining all controls. The aim was to reduce within-case heterogeneity and limit attenuation of case–control differences introduced by samples with control-like transcriptional profiles. Restricting the analyses to symptomatic individuals increased the number of DEGs and strengthened effect sizes, while preserving the direction of the main signals seen in the all-inclusive analyses. In CCD (symptomatic vs. controls), we confirmed what was previously observed. In MmD, the symptomatic-only analyses yielded more DEGs than the inclusive analysis; however, confirming *ARHGAP36* upregulation and *ATN1* and *PAIP2* down-regulation, pointing to the active gene regulation needed to control the mitochondrial network and to maintain mitochondrial and cellular health. To our surprise, *MYL12A* emerged as a down-regulated gene. At the pathway level, the symptomatic-only re-analysis amplified enrichment magnitudes rather than changing their nature. In the CCD versus control comparison, GO-ORA broadened yet remained dominated by muscle-structure and contractile programs; the oxidative-stress branch (responses to ROS and H_2_O_2_) persisted, whereas SMAD signal transduction no longer reached significance. It is difficult to determine the reasons for the reduced significance of SMAD signalling in the symptomatic analyses; this finding may suggest that the pathway is more relevant during the early stages of disease progression rather than in advanced stages. Unfortunately, longitudinal muscle biopsies from the same patients are not available to directly test this hypothesis. However, a transition in the histopathological phenotype of CCD has been well documented [92], likely suggesting profound changes also in dysregulated genes. The symptomatic MmD versus control confirmed previous findings. A caveat remains in this analysis: restricting the dataset to symptomatic cases inevitably reduces the sample size, which may limit statistical power. As a result, the subtle transcriptomic changes detected in this subset likely reflect a larger underlying effect size and lower expression variability, rather than the identification of novel or unexpected gene dysregulation. Nevertheless, we consider the symptomatic-only results to be corroborative of the broader analyses. Importantly, the symptomatic-only approach appears to enrich for disease-proximal molecular signals, providing a clearer view of pathways most directly associated with clinical manifestation. In contrast, the inclusive analyses, encompassing both symptomatic and minimally affected individuals, likely capture the wider molecular landscape of the disease, including adaptive, compensatory, or subclinical expression profiles. Together, these complementary perspectives underscore the continuum of molecular changes underlying disease progression and highlight the value of integrating both focused and inclusive transcriptomic approaches to disentangle pathogenic from adaptive responses.

## 5. Conclusions

Transcriptome-wide profiling of RYR1-RM (CCD and MmD) reveals that disease mechanisms extend beyond calcium dysregulation to encompass mitochondrial dysfunction, altered SMAD-dependent dynamics, and remodelling of translational and stress-response pathways. CCD is marked by strong perturbations in muscle structural and mitochondrial genes, while MmD shows subtle but convergent changes linking metabolic and immune-related processes. Analyses restricted to symptomatic patients highlight disease-proximal molecular signals, underscoring the continuum between adaptive and pathogenic responses. Collectively, these findings establish a comprehensive molecular framework for RYR1-RM and provide a foundation for future mechanistic and therapeutic investigations. Exploring RNA-seq in other diseases due to variants in other calcium homeostasis genes could provide further insights into the pathways activated by calcium dysregulation and may help identify gene-specific signatures. It is important to note that while RNA-seq enables comprehensive quantification of transcriptomic alterations, differences in DEGs should always be interpreted with caution in terms of biological significance. Changes, even when they reach statistical significance, may also reflect technical variability (including muscle tissue heterogeneity, sampling differences, or transient physiological states) rather than primary disease mechanisms. Moreover, modest transcriptomic alterations may not correspond to proportional changes at the protein or functional level due to post-transcriptional regulation and compensatory adaptive responses. These limitations are intrinsic to RNA-seq approaches; therefore, all findings should be confirmed by cell-based experiments before they can be considered robust evidence.

## Figures and Tables

**Table 1:** Patients included in the study.

Sample ID	Sex	Age at Biopsy	Nucleotide Change	Amino Acid Change	Exon	Histological Diagnosis	Protein Domain	Structural Effect	Disease Severity	References
ID01	M	55	c.14209C>T	p.Arg4737Trp	98	MmD	pVSD	Impairs a salt bridge with Glu4736. Impairs apoCAM binding.	Mild myopathy	[34,35]
ID02	M	53	c.7048G>A	p.Ala2350Thr	44	CCD	Bsol	New interaction with Ser2345. Loss of interaction with calstabin 1.	Mild myopathy	[36]
ID03	M	35	c.6617C>T	p.Thr2206Met	40	MmD	Bsol	Hydrophobic residue inside a polar pocket. Increases caffeine sensitivity.	Asymptomatic	[37]
			c.10537A>G	p.Thr3513Ala	71		Bsol	Hydrophobic residue inside a polar pocket. Impairs apoCAM binding.		[3]
ID04	M	24	c.467G>A	p.Arg156Lys	6	CCD	NTD-A	Impairs an exposed salt bridge with Glu160. Destabilises MIR folding domain.	Paucisymptomatic	[38,39]
ID05	M	33	c.14209C>T	p.Arg4737Trp	98	MmD	pVSD	Impairs a salt bridge with Glu4736. Impairs apoCAM binding.	Paucisymptomatic	[34,35]
ID06	F	41	c.3901C>T	p.Arg1301Cys	28	MmD	SPRY2/SPRY3	No local modification. Interferes with DHPR regulation.	Asymptomatic	dbSNP: rs745920741
			c.5360C>T	p.Pro1787Leu	34		Jsol	No local modification. Likely benign.		[39]
ID07	F	46	c.7304G>A	p.Arg2435His	45	CCD	Bsol	Weaker electrostatic interaction with Asp2431. Inactivates the channel gating.	Paucisymptomatic	[37]
ID08	F	29	c.7523G>A	p.Arg2508His	47	CCD	Bsol	Impairs a salt bridge with Glu42439. Loss of interaction with calstabin.	Paucisymptomatic	[38,40]
ID09	F	33	c.487C>T	p.Arg163Cys	6	CCD	NTD-A	Impairs electrostatic interactions with Glu160 and Glu2088. Destabilises MIR folding domain.	Paucisymptomatic	[41,42]
ID11	F	37	c.487C>T	p.Arg163Cys	6	CCD	NTD-A	Impairs electrostatic interactions with Glu160 and Glu2088. Destabilises MIR folding domain.	Moderate myopathy	[41,42]
ID12	F	41	c.14510delA	p.Gln4837ArgfsX3	100	CCD	pore	Possible local misfolding. Alters quantitative level of RyR1.	Mild myopathy	[39]
ID13	F	22	c.7085A>G	p.Glu2362Gly	44	CCD	Bsol	Putative loss of interaction with ions. Inactivates the channel gating.	Moderate myopathy	[38]
			c.13513G>C	p.Asp4505His	92		pVSD	Possible local unfolding. Impairs apoCAM binding.		[43,44]
ID14	M	6	c.4711A>G	p.Ile1571Val	33	MmD	RY2/SPRY3	Hydrophobic residue inside a polar pocket. Likely benign.	Paucisymptomatic	[45,46]
			c.9407delT	p.Leu3136ArgfsX3	63		Bsol	Possible local misfolding. Alters quantitative level of RyR1.		[3]
ID15	F	12	c.14693T>C	p.Ile4898Thr	102	CCD	pore	Polar residue inside a hydrophobic pocket. Disrupts the channel activity.	Moderate myopathy	[27,47]
ID16	F	12	c.472_474delGAA	p.Glu158del	6	CCD	NTD-A	Possible local misfolding. Reduces MIR2 folding domain.	Severe myopathy	[3]
ID17	M	3	-	-	-	Ctrl	-		-	
ID18	M	8	-	-		Ctrl	-		-	
ID19	F	6	-	-		Ctrl	-		-	
ID20	M	37	-	-		Ctrl	-		-	
ID21	F	35	-	-		Ctrl	-		-	
ID22	M	57	-	-		Ctrl	-		-	
ID23	F	30	-	-		Ctrl	-		-	

Ctrl, control; CCD, central core disease; MmD, multi-minicore disease; M, male; F, female.

## Data Availability

The datasets supporting the conclusions of this article are included within the article (and its Supplementary Files).

## Supplementary Materials

The following supporting information can be downloaded at: https://www.mdpi.com/article/10.3390/biom15111599/s1, Figure S1: Schematic representation of the human RYR1 protein domains and distribution of variants identified in this study. The linear map illustrates the main structural and functional domains of the human RYR1, including regions involved in DHPR and CaM binding, MH/CCD hotspots, and the ion transport domain. Coloured boxes indicate key interaction sites and functional motifs, while the positions of variants detected in this study are shown below. Variants are clustered within regions associated with excitation–contraction coupling, calcium binding, and channel selectivity. In the upper bar, the relative positions of the major RYR1 domains, including SPRY, RYR, and IP3 receptor-related regions, as well as the ryanodine receptor and ion transport segments, are presented. Figure S2: Summary of the RNASeq analysis workflow. Following alignment using STAR and quantification, transcript information was collapsed to the gene level, facilitated by tx2gene obtained via the ‘AnnotationHub()’ function. DESeq was employed for differential expression analysis, and successful normalisation was confirmed through the relative log expression plot ‘plotRLE()’. Pathway analysis (overrepresentation analysis) was conducted using DESeq2 results with Gene Ontology terms, and a Gene Set Enrichment Analysis was performed with KEGG terms (for further details, refer to the text below). Figure S3: Principal component analysis (PCA) plots comparing CCD and controls (A) and MMD and controls (B). Figure S4: Validation by qRT-PCR. Analysis of the transcript levels of the indicated genes normalised to beta-actin transcript levels in the CCD muscle compared with controls (*n * =  5 MmD, *n* = 6 Ctrl). Figure S5: Volcano plot showing the results of differential expression analysis of RYR1-RM patients vs. controls using DESeq2. Each dot represents a gene, plotted according to its log2FC on the X-axis and the −log10 adjusted *p*-value on the Y-axis. Blue dots indicate DEGs (*p*-adj < 0.05 with a log2FC ≥ 0.58 or ≤−0.58). DEGs were labeled. Figure S6: Volcano plot showing the results of differential expression analysis of symptomatic RYR1-RM patients vs. controls using DESeq2. Each dot represents a gene, plotted according to its log2FC on the X-axis and the −log10 adjusted *p*-value on the Y-axis. Blue dots indicate DEGs (*p*-adj < 0.05 with a log2FC ≥ 0.58 or ≤−0.58). DEGs were labeled (only a subset of the most dysregulated genes was shown for readability). Figure S7: Volcano plot showing the results of differential expression analysis of symptomatic CCD patients vs. controls using DESeq2. Each dot represents a gene, plotted according to its log2FC on the X-axis and the −log10 adjusted *p*-value on the Y-axis. Blue dots indicate DEGs (*p*-adj < 0.05 with a log2FC ≥ 0.58 or ≤−0.58). DEGs were labeled (only a subset of the most dysregulated genes was shown for readability). Figure S8: Volcano plot showing the results of differential expression analysis of symptomatic MmD patients vs. controls using DESeq2. Each dot represents a gene, plotted according to its log2FC on the X-axis and the −log10 adjusted *p*-value on the Y-axis. Blue dots indicate DEGs (*p*-adj < 0.05 with a log2FC ≥ 0.58 or ≤−0.58). DEGs were labeled (only a subset of the most dysregulated genes was shown for readability). Figure S9: RYR1-RM patients vs. controls dotplots: (A) dotplot showing the Gene Ontology (GO) terms according to the gene ratio (DEGs related to a GO term/total number of genes of the GO term). The colour represents the *p*-value adjusted according to the HB method (*p*-adj, as in the legend), and the dot’s size indicates the number of genes involved in each pathway. (B) Dotplot showing existing relationships among the significantly enriched GO terms (*p*-adj) resulting from over-representation analysis. Figure S10: RYR1 vs. controls: relationships occurring between genes associated with the significant GO terms and relative fold change (colour). The size of the dots indicates the significance of the GO terms. Figure S11: Symptomatic CCD vs. controls dotplots: (A) dotplot showing the top 50 Gene Ontology (GO) terms according to the gene ratio (DEGs related to a GO term/total number of genes of the GO term). The colour represents the *p*-value adjusted according to the HB method (*p*-adj, as in the legend), and the dot’s size indicates the number of genes involved in each pathway. (B) Dotplot showing existing relationships among the 50 most significant enriched GO terms (*p*-adj) resulting from over-representation analysis. For graphical reasons, not all labels corresponding to the muscle function and muscle physiology macrocategory are displayed. Figure S12: Symptomatic CCD vs. controls: relationships occurring between genes associated with the 20 most significant GO terms and relative fold change (colour). The size of the dots indicates the significance of the GO terms. Figure S13: Symptomatic MmD vs. controls dotplots: (A) dotplot showing the top 50 Gene Ontology (GO) terms according to the gene ratio (DEGs related to a GO term/total number of genes of the GO term). The colour represents the *p*-value adjusted according to the HB method (*p*-adj, as in the legend), and the dot’s size indicates the number of genes involved in each pathway. (B) Dotplot showing existing relationships among the 50 most significant enriched GO terms (*p*-adj) resulting from over-representation analysis. For graphical reasons, not all labels corresponding to the muscle function and muscle physiology macrocategory are displayed. Figure S14: Symptomatic MMD vs. controls: relationships occurring between genes associated with the 20 most significant GO terms and relative fold change (colour). The size of the dots indicates the significance of the GO terms. Figure S15: Symptomatic RYR1 vs. controls dotplots: (A) dotplot showing the Gene Ontology (GO) terms according to the gene ratio (DEGs related to a GO term/total number of genes of the GO term). The colour represents the *p*-value adjusted according to the HB method (*p*-adj, as in the legend), and the dots’ size indicates the number of genes involved in each pathway. (B) Dotplot showing existing relationships among the significantly enriched GO terms (*p*-adj) resulting from over-representation analysis. Figure S16: Symptomatic RYR1 vs. controls: relationships occurring between genes associated with the significant GO terms and relative fold change (colour). The size of the dots indicates the significance of the GO terms. Table S1: List of primers. Table S2: Patients’ RYR1 clinical description. Table S3: CCD vs. controls. Table S4: CL5 CCD vs. controls. Table S5: MmD vs. controls. Table S6: All RYR1-RM vs. controls. Table S7: Symptomatic RYR1-RM vs. controls. Table S8: Symptomatic CCD vs. controls. Table S9: Symptomatic MmD vs. controls. Table S10: CCD vs. controls ORA. Table S11: CCD vs. controls GSEA. Table S12: MmD vs. controls GSEA. Table S13: All RYR1-RM vs. controls ORA. Table S14: All RYR1-RM vs. controls GSEA. Table S15: Symptomatic CCD vs. controls ORA. Table S16: Symptomatic CCD vs. controls GSEA. Table S17: Symptomatic MmD vs. controls ORA. Table S18: Symptomatic MmD vs. controls GSEA. Table S19: Symptomatic RYR1-RM vs. controls ORA. Table S20: Symptomatic RYR1-RM vs. controls GSEA. Table S21: Summary of the discussed pathways/GO terms identified as significantly enriched in the ORA analyses performed on all differentially expressed genes (All DEGs) and separately on up- or down-regulated DEGs. Table S22: Side-by-side comparison of selected pathways across CCD, MmD, and RYR1 vs. controls GSEA analyses.

## Abbreviations

*RYR1*-RM	Ryanodine Receptor 1-Related Myopathies
CCD	Central Core Disease
MmD	Multi-Mini Core Disease
MAPK	Mitogen-Activated Protein Kinase
ZFP36	Zinc Finger Protein 36
ATN1	Ataxin 1
DuCD	Dusty Core Disease
SR	Sarcoplasmic Reticulum
GLM	Generalised Linear Model
log2FC	Log2FoldChange
HB	Benjamini and Hochberg
ORA	Over-Representation Analysis
GO	Gene Ontology
DE	Differential expression
GEA	Gene Enrichment Analysis
DEGs	Differentially Expressed Genes
PCA	Principal Component Analysis
MYL12A	Myosin Light Chain 12A
TPPP3	Tubulin Polymerisation Promoting Protein Family Member 3
BEX2	Brain Expressed X-Linked 2
SOCS3	Cytokine Signalling 3
GADD45B	Growth Arrest and DNA Damage Inducible Beta
CL5 CCD	Clustered Group of 5 CCD
PAIP2	Poly(A) Binding Protein Interacting Protein 2
ATN1	Atrophin 1
ARHGAP36	Rho Gtpase Activating Protein 36
GBP1	Guanylate-Binding Protein 1
GBP4	Guanylate-Binding Protein 4
TGF-β	Transforming Growth Factor-β
UPR	Unfolded Protein Response
ER	Endoplasmic Reticulum
SOCS3	Suppressor Of Cytokine Signalling 3
GRP78	Glucose Regulatory Protein 78
AATF	Antiapoptotic Transcription Factor
MH	Malignant Hyperthermia
