# Supplementary figures and images for: RYR1-Related Myopathies Involve More than Calcium Dysregulation: Insights from Transcriptomic Profiling
Source: Biomolecules. 2025 Nov 14;15(11):1599. doi: 10.3390/biom15111599 (PMC12650710; doi:10.3390/biom15111599)

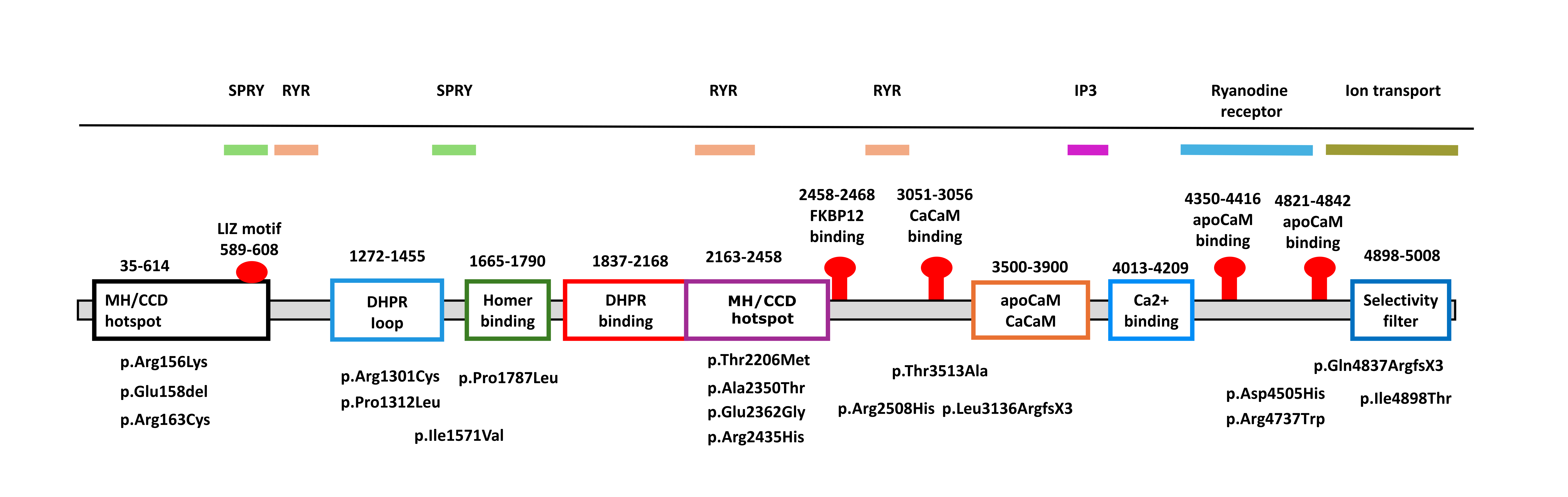

Supplement: Supplementary file 1 [file biomolecules-15-01599-s001.zip › Figure_S1.png]

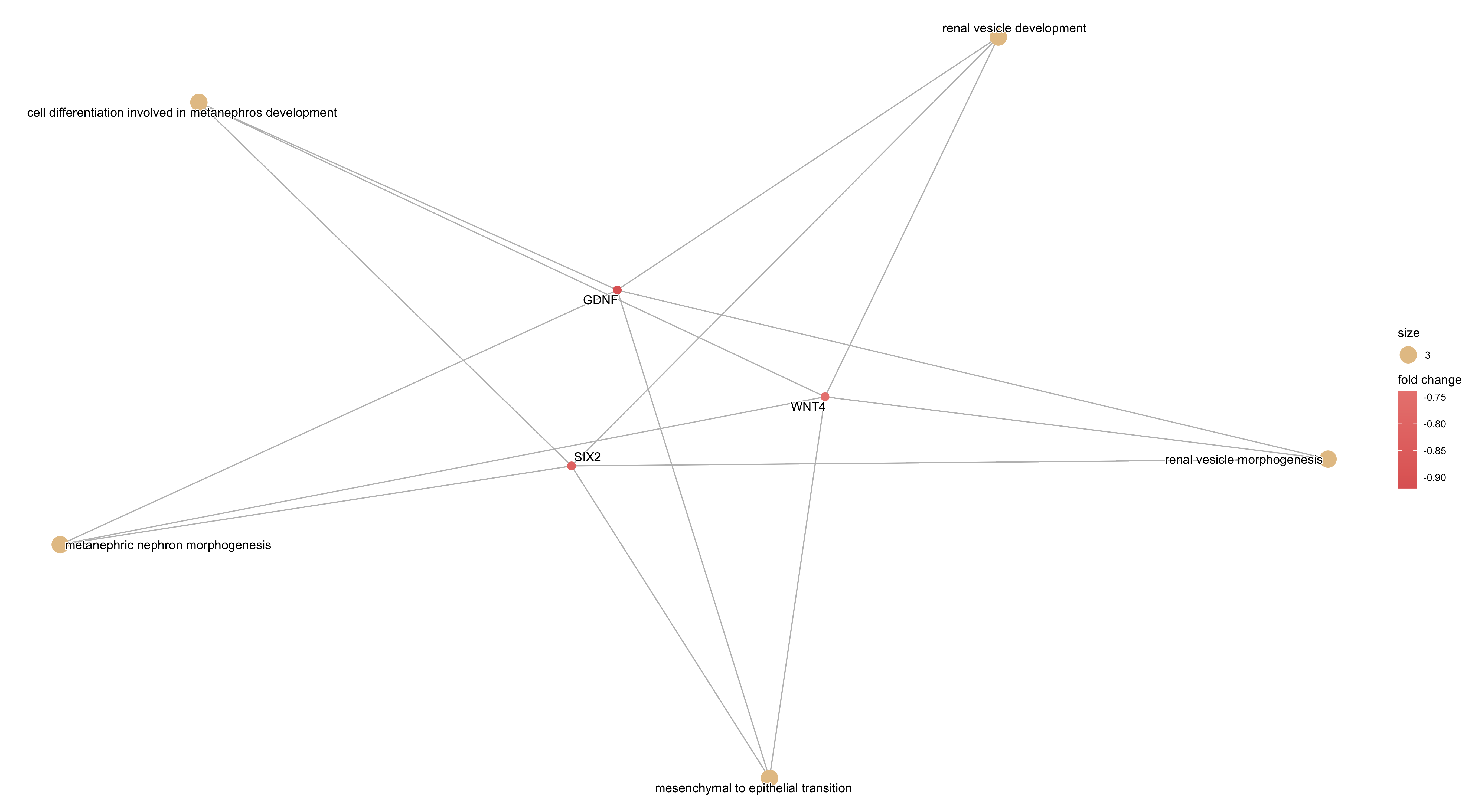

Supplement: Supplementary file 1 [file biomolecules-15-01599-s001.zip › Figure_S10.png]

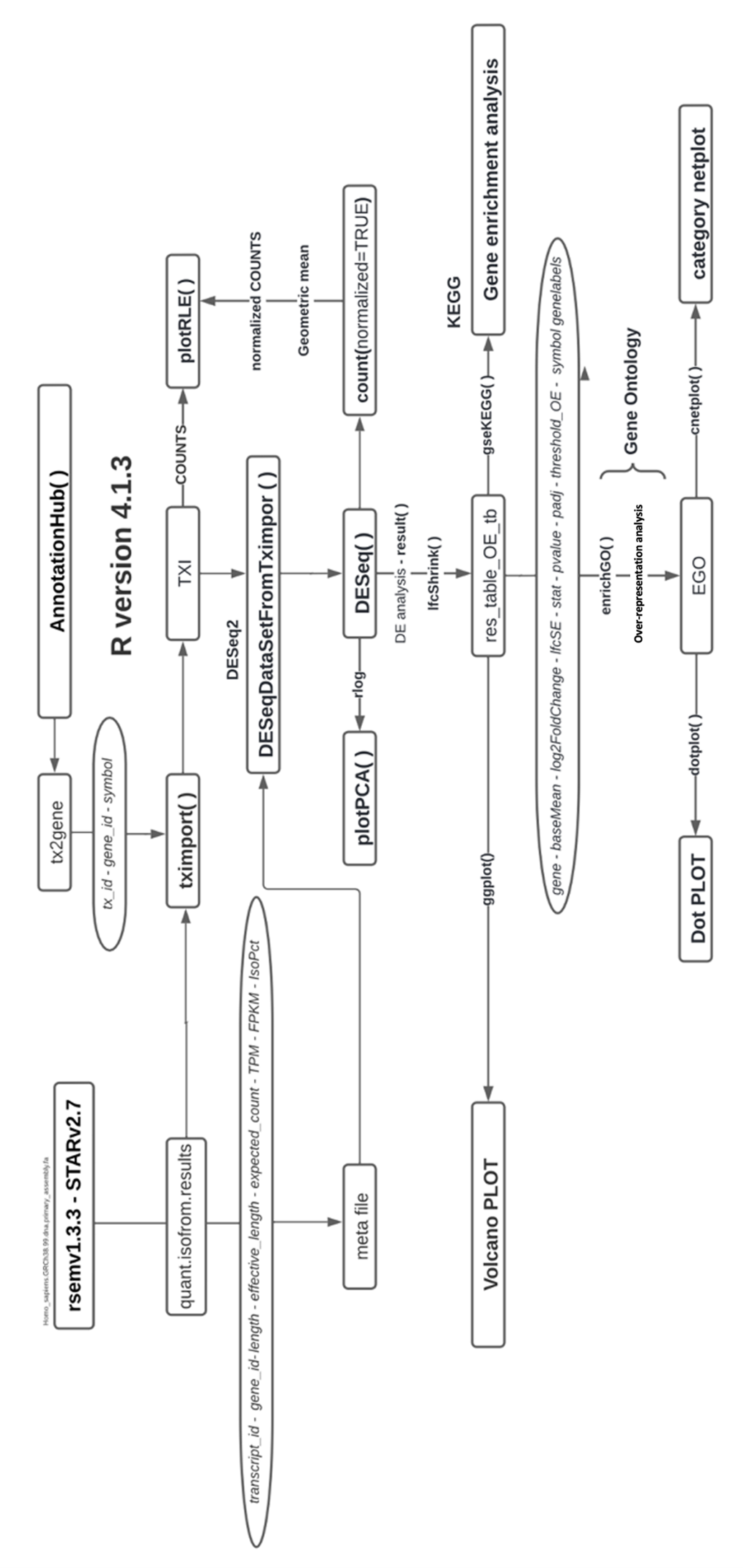

Supplement: Supplementary file 1 [file biomolecules-15-01599-s001.zip › Figure_S2.png]

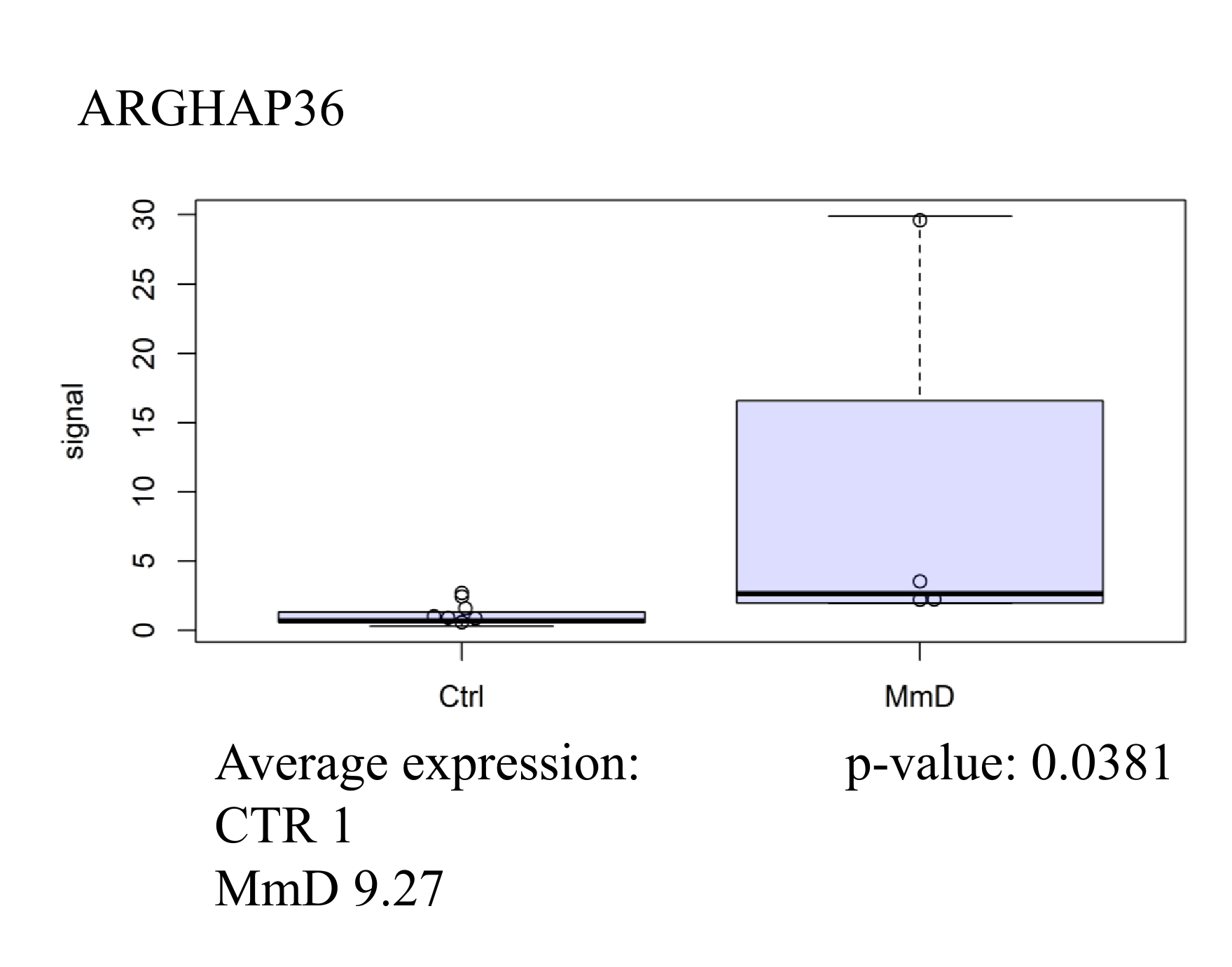

Supplement: Supplementary file 1 [file biomolecules-15-01599-s001.zip › Figure_S4.png]
